# Prevalence and Associated Factors of Self-Reported Gingival Bleeding: A Multicenter Study in France

**DOI:** 10.3390/ijerph17228563

**Published:** 2020-11-18

**Authors:** Thomas Veynachter, Valérie Orti, Estelle Moulis, Hélène Rousseau, Nathalie Thilly, Fani Anagnostou, Sylvie Jeanne, Catherine Bisson

**Affiliations:** 1 Faculty of Dentistry, University of Lorraine, 54505 Vandœuvre-lès-Nancy, France; catherine.bisson@univ-lorraine.fr; 2 Department of Periodontology, University Hospital, 54000 Nancy, France; 3 SIMPA, Stress Immunity Pathogens Unit, Brabois Santé Campus, EA 7300, F-54000 Nancy, France; 4 Department of Dentistry, University of Montpellier, 34193 Montpellier, France; valerie.orti@umontpellier.fr (V.O.); estelle.moulis@umontpellier.fr (E.M.); 5 Platform Support for Clinical Research, University Hospital, 54505 Vandoeuvre-Lès-Nancy, France; h.rousseau@chru-nancy.fr (H.R.); n.thilly@chru-nancy.fr (N.T.); 6 Department of Dentistry, Garancière, University of Paris Diderot, 75006 Paris, France; fani.anagnostou@univ-paris-diderot.fr; 7 Department of Periodontology, University of Rennes, 35043 Rennes, France; sylvie.jeanne@univ-rennes1.fr

**Keywords:** self-reported gingival bleeding, anxiety level, socioeconomic level, toothbrushing technique, drugs

## Abstract

Gingival bleeding (GB) is a common sign of gingival inflammation which indicates the presence of periodontal diseases. This cross-sectional multicenter survey aimed to assess the prevalence of self-reported gingival bleeding (SRGB) in French adults and identify the main associated factors. A questionnaire-based interview was randomly proposed to 794 individuals in four French cities (Nancy, Montpellier, Paris, and Rennes). Subjects were recruited in preventive medicine centers (50%), railway stations, and malls (50%). The questionnaire comprised 25 items: SRGB characteristics, socioeconomic variables, oral hygiene habits, use of drugs, and anxiety level. The overall prevalence of SRGB was 63.2% [59.8%; 66.6%], with 58.7% bleeding after toothbrushing and 4.5% spontaneous bleeding. Males reported significantly lower SRGB prevalence than females (*p* = 0.04). The distribution of SRGB frequency was inversely proportional to age (*p* < 0.0001). No association between drug use and SRGB was found. The people interviewed in the preventive medicine centers reported the highest frequency of SRGB (*p* < 0.0001). In the multivariate logistic model, SRGB was significantly related to occupation, smoking status, brushing frequency, and anxiety level. In conclusion, SRGB was prevalent in more than half of the sample and was mainly associated with age, toothbrushing frequency, and anxiety level. Thus, providing information to patients about the importance of this oral manifestation may play an important role in preventing periodontal diseases.

## 1. Introduction

Gingival bleeding (GB) is one of the first clinical manifestations of periodontal diseases such as gingivitis and periodontitis. This bleeding is the sign of gingivitis, which is considered a reversible form of periodontal disease when only the gingiva is affected but may turn into periodontitis for certain patients if left untreated. Severe periodontitis is the sixth most prevalent disease in the world, and it is the main cause of disability-adjusted life years among oral conditions because of the tooth loss potentially induced by this pathology [1]. Thus, GB can be considered as warning sign for the patient that should prompt them to consult a dentist. Bleeding is one of the most reliable parameters in evaluating periodontal status. According to the new classification of periodontal diseases [2], the percentage of sites with bleeding on probing (BoP), assessed during a basic periodontal examination, provides information on the presence of gingivitis (BoP > 10%) and is considered in the periodontal risk assessment for patients in supportive periodontal therapy, as described by Lang and Tonetti [3]. Bleeding can appear after toothbrushing or on probing, or it may be spontaneous. Self-reported gingival bleeding (SRGB) may provide significant information about periodontal diseases and periodontal treatment needs in a population.

Gingivitis is an inflammatory lesion of the marginal gingiva that can result from a combination of local factors, but it is mainly due to microbial plaque build-up on the teeth and can be enhanced by general factors (genetics, systemic disorders, and medications). Drugs can modulate hemostasis, vascular permeability, and coagulation. Environmental, behavioral, or sociodemographic factors (age, gender, stress, smoking, or socioeconomic status) may also influence the onset and evolution of gingival inflammation [4,5,6]. As shown in an observational French pharmacovigilance database study, GB can be a serious adverse drug reaction reported by health care professionals or patients. Spontaneous GB not related to periodontal disease is associated in particular with antithrombotics and to a lesser extent with antibacterial and psychoanaleptic drugs [7].

Very few studies have reported the periodontal status of the general population in France. In 1993 and 1995, a regional study was carried out among 35–44- and 65–74-year-old adults, respectively: 59.2% of the former and 11.6% of the latter demonstrated GB, detected after a professional periodontal examination [8,9]. In 2007, a French national survey showed that 50% of the population had severe clinical attachment loss, while more than 80% showed probing depth pockets >2 mm, but GB was not assessed [10]. There are no recent data about the periodontal status of the French population. Even if the clinical examination is usually considered the gold standard in diagnosis and assessment of periodontal status, SRGB may be an alternative and relevant method in simplifying the periodontal data-collection process in evaluating the periodontal condition and treatment needs in public health [11,12]. Indeed, for certain authors, self-reporting is an efficient means of assessing many population characteristics, risk factors, and pathologies, but it has rarely been used for periodontal disease. Recently, Carra et al. developed a periodontal screening score which represents, according to the authors, a valuable and accurate tool in detecting severe periodontitis (but not gingivitis) using a self-reported assessment of the population [13]. More recently, Saka Herrán et al. developed a new questionnaire, including questions on GB, capable of evaluating periodontitis with good precision [14]. To our knowledge, no studies using self-reported gingival bleeding have been carried out in France to assess gingivitis.

The aim of this cross-sectional multicenter survey was first to assess the proportion of subjects experiencing GB, and then to identify the main factors linked to GB by means of a conducted questionnaire.

## 2. Materials and Methods

This nationally representative cross-sectional study is reported according to the Strengthening the Reporting of Observational Studies in Epidemiology (STROBE) guidelines [15,16].

### 2.1. Study Design

This cross-sectional multicenter survey was conducted between September 2016 and November 2017 in four cities in France. A questionnaire-based interview was randomly proposed to people encountered in three different sites in each selected city (preventive medicine centers, railway stations, and malls). The ratio distribution was 50% in the preventive medicine centers and 50% in the public spaces (25% in railway stations and 25% in malls). The preventive medicine centers are organizations where all French people, regardless of socioeconomic status, age, or sex, have their health status evaluated. Consequently, this ratio was more representative than an equal distribution in the three centers. The cities (Paris, Rennes, Nancy and Montpellier) were selected because they are located in four different parts of France (north, west, east, and south) according to the French National Institute for Statistics and Economic Studies [17] and because they all had a dental school.

### 2.2. Interview

In each city, one student from the dental school conducted face-to-face interviews lasting about 5 min in the three locations (preventive medicine center, railway station, and mall).

The questionnaire consisted of 25 items, including socioeconomic status, tobacco use, toothbrushing habits, anxiety level, and type of bleeding. The primary endpoint was the prevalence of GB reported by the subjects who took part in the survey. More details are provided in Appendix A.

The ethics committee of the Nancy CHRU Hospital gave a favorable opinion regarding this interview (Saisine No. 282).

### 2.3. Statistical Methods

The calculation of the sample size is detailed in Appendix B. 

Percentages and proportions were drawn from the different items of the questionnaire, and the GB prevalence was calculated accordingly. Chi-square tests were performed in bivariate analyses between GB and its potential risk factors. A stepwise multiple logistic regression (respectively, a stepwise multinomial logistic regression) was used to determine factors associated with GB (there were three modalities: spontaneous GB, toothbrushing GB, and absence of GB) among covariates, with a *p*-value less than 0.20 in the bivariate analyses. All analyses were performed with the statistical software SAS version 9.4 (SAS Institute, Inc., Cary, NC, USA) and the significance level was set at 0.05.

## 3. Results

In total, 794 questionnaires (200 in Montpellier, 200 in Nancy, 200 in Paris, and 194 in Rennes) were completed correctly.

### 3.1. Characteristics of the Population

Table 1 presents the demographics and information on toothbrushing in the interviewed population by center. The distribution of age, smoking status, educational level, and occupation of the interviewees was statistically different between preventive medicine (PM) centers and railway stations or malls (*p* = 0.039, *p* = 0.003, *p* = 0.0001, and *p* = 0.001, respectively). People interviewed in PM centers presented a greater number of smokers than those in the other places (i.e., railway stations and malls). No difference between the sex of the interviewees in PM and other centers was observed. More details are provided in Appendix C. A full description of the characteristics of people included in the four French cities is given in Table S1. 

### 3.2. Self-Reported Prevalence of Gingival Bleeding

**Figure 1:**
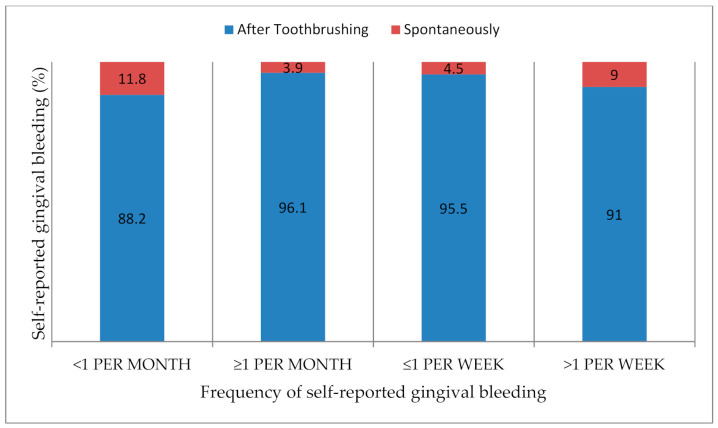
Description of the type of self-reported gingival bleeding according to frequency.

The overall prevalence of SRGB was 63.2% [59.8%; 66.6%], with 4.5% reporting spontaneous bleeding and 58.7% reporting bleeding after toothbrushing. Among those reporting GB, 22.1% reported bleeding appearing less than once a week, and 19.9% reported bleeding appearing more than once a week (Table 2). Regardless of how often bleeding appeared (less than once per month, more than once per month, less than once per week, or more than once per week), SRGB was mainly found after toothbrushing rather than spontaneously (88.2% vs. 11.8%, 96.1% vs. 3.9%, 95.5% vs. 4.5%, and 91.0% vs. 9.0%, respectively) (Figure 1). Among women reporting GB, only 18.7% (52/278) mentioned hormonal gingival bleeding (during pregnancy, menstrual bleeding, and the use of contraceptive pills) (Table 2). The description of SRGB frequency in the different cities is available in Appendix D. 

Regarding the overall prevalence of SRGB, the report of bleeding was statistically different depending on the location of the interview; regardless of the city, the highest prevalence was observed in PM centers (*p* < 0.0001) (Table 3). By contrast, no significant difference was observed regarding the distribution of the frequency of SRGB (less than once per month, more than once per month, less than once per week, or more than once per week) in all centers (Table 3).

**Figure 2:**
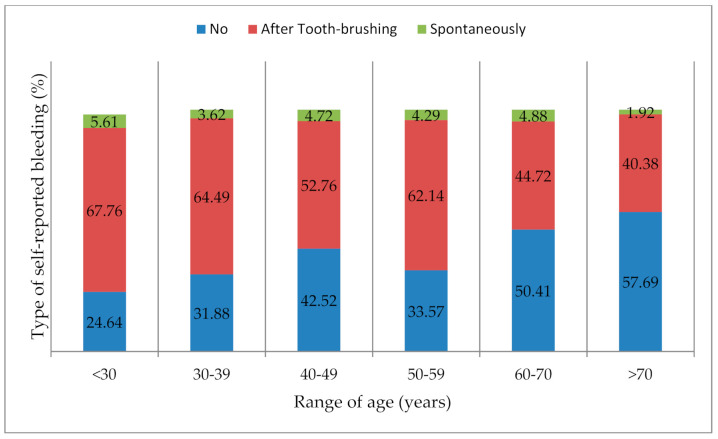
Frequency of the types of self-reported gingival bleeding according to range of age.

Among all participants, 59.6% of men and 66.5% of women reported GB. This difference of SRGB prevalence was significant (*p* = 0.04) (Table 4), but after stratification by age, no significant difference was observed in regards to sex (data not shown). Regarding the overall SRGB data, the distribution of its prevalence was inversely proportional to age (*p* < 0.0001) for both spontaneous and toothbrushing-associated bleeding (Table 4, Figure 2).

### 3.3. Factors Associated with Self-Reported Gingival Bleeding

#### 3.3.1. Relationship between GB and Socioeconomic, Medical, and Dental Variables

Regarding the socioeconomic status of the participants, the distribution of the occupational status (but not of the educational level) was significantly associated with SRGB (*p* < 0.0001) (Table 4). Retired people and categories such as teachers, liberal professions, and executives presented the lowest frequency of SRGB. No association was observed between SRGB and medications, except for anxiolytics and antidepressants (*p* = 0.067). No significant differences were observed between smokers and nonsmokers regarding SRGB, even if the percentage of SRGB was lower in people smoking more than 10 cigarettes per day. By contrast, the distribution of anxiety levels was significantly associated with SRGB (*p* = 0.0004). Those with high or severe anxiety presented the highest frequency of SRGB.

#### 3.3.2. Relationship between Self-Reported GB and Toothbrushing Variables

Only the frequency of toothbrushing was statistically correlated with the prevalence of gingival bleeding (*p* < 0.025) (Table S2). More details about these variables are available in Appendix E.

#### 3.3.3. Factors Associated with Self-Reported GB

Independent variables with a *p*-value < 0.20 in the bivariate analyses were likely to enter the multivariate model. The age variable was withdrawn from the model because of its correlation with the occupation variable. 

Factors associated with SRGB were occupation, smoking status, toothbrushing frequency, and anxiety level. The occupations classes none, unemployed, student, other or manual worker, employee, artisan, retailer, and administrative (*p* < 0.0001); toothbrushing less than two times per day (*p* = 0.0047); and a high/severe anxiety level (*p* = 0.0031) were associated with a higher risk of GB. In contrast, smoking was associated with a lower risk of SRGB (*p* = 0.0012) (Table 5). 

The multinomial logistic regression model, including three modalities for GB (spontaneous SRGB, toothbrushing SRGB, absence of SRGB), confirmed the results of the multiple logistic regression model, with differences detected only between toothbrushing SRGB and absence of SRGB, due to fewer events of spontaneous SRGB (Table 6).

## 4. Discussion

This study is the first self-evaluation of gingival bleeding in France. Among responders, the overall prevalence of SRGB was 63.2%, regardless of age and circumstances of onset. In the literature, a wide range of SRGB prevalence has been published. In different studies of self-reported periodontal health, from 6 to 78% of responders reported GB [18,19]. Pinelli et al. evaluated self-perceived oral health conditions and concluded that among the 200 Brazilians interviewed, 53.5% declared GB after toothbrushing and 6% declared spontaneous GB, which is in accordance with our results. Furthermore, Genco et al. found similar results for the question “bleeding gums in the past”; 53% of interviewees in the Erie County Study answered positively [20]. The SRGB frequency we found is in accordance with the results of the French clinical epidemiologic study evaluating GB with Community Periodontal Index of Treatment Needs (CPITN), which underlines the fact that gingival inflammation has not decreased in the French population for 23 years [8,9]. The large diversity in results might be explained by the difference in length of the questionnaires. Indeed, people respond less easily to long questionnaires than short ones [21,22]. Moreover, the many different ways of collecting data that explore gingival health [12,23], the different methodologies of evaluating gingival health (swollen or bleeding gingiva, gingival disease or inflammation) [24,25], and population-characteristic differences [26,27] make the comparison of results difficult.

Apart from the Gilbert and Nuttall study with the highest score of SRGB (78% of British adult patients in dental hospitals), all the papers described lower percentages of SRGB than our results [19]. In the Dietrich cohort, 44% of people reported GB after toothbrushing [24]. The same percentage was reported in the Yamamoto cohort of men aged 50 to 59 [26]. The lowest percentages of SRGB were observed in the Saka-Herrán, Lintula, Kim, and Taylor studies: 32% of the Spanish interviewed, 24% of the 31-year-old Finnish, 11.3% of selected Koreans having GB after toothbrushing, and 17% of Americans interviewed presented GB in the previous week, respectively [14,23,28,29]. The differences in age groups, gender ratios, educational levels, occupations, and the percentages of smokers included in the cohorts could explain the discrepancies with the results of our study.

Previous epidemiological studies have demonstrated the age dependence and gender differences of gingival bleeding data [8,9,28,30]. Our results found a higher prevalence of GB among females and are in line with those of other studies [8,28,31]. The higher SRGB prevalence observed in females could be attributed to the facts that (i) women are more aware of their oral health and (ii) women are more susceptible to gingival inflammation exacerbations due to hormonal fluctuations [32,33]. In contrast to our results, Kim et al. observed similar SRGB prevalence between males and females, but no information regarding smoking was available and the subjects of the survey were slightly older [29]. Our study showed a significant decrease in SRGB frequency with age; a lower percentage was reported by people aged 60 and over, which is in accordance with the conclusions of Kim [29]. Two French clinical epidemiologic studies evaluated GB with the CPITN and showed similar results for each age group evaluated [8,9]. By contrast, Buhlin et al. observed an inverse association of SRGB according to age [34]. Elderly people have a higher number of missing teeth than those younger, but most importantly, they seem to be less aware of their gingival bleeding, as shown in the study of Romano et al. [31]. Ebersole et al. demonstrated the presence of age-associated alterations in innate immune function within the periodontium, which could affect not only the initiation but also the resolution of inflammation [35]. This phenomenon may modulate the GB of aging people.

The relationship between socioeconomic status (SES) and general health, including oral health and involving different mechanisms, has often been explored [36]. Individuals with low SES are more likely to be affected by oral disease [37]. Although SRGB prevalence was significantly higher among unemployed people in the bivariate analysis, we could not highlight any difference in SRGB prevalence according to occupational status in the multivariate model after adjusting for the other covariates. Since occupational status is only a limited aspect of SES, this result would need further analyses, such as those incorporating data about household income, to be confirmed. The prevalence of SRGB was statistically different depending on the centers. Interviewees in preventive medicine centers presented the highest frequency of SRGB, probably because they were more aware of their periodontal health than the others. Indeed, these interviewees were present in these centers either because they were having a health check-up and/or because they were included in national health surveys on different diseases.

Many authors have reported that medications may cause GB through an effect on vascular permeability, coagulation, or platelet function [38,39]. A French pharmacoepidemiologic study analyzed GB as a possible serious adverse drug reaction and showed that GB represented only 0.09% of all reported adverse drug reactions and is more commonly associated with an increased international normalized ratio (INR), thrombocytopenia, or hematuria [7]. In our study, no significant association was found between SRGB and any of the drugs evaluated, but it is interesting to note that few participants reported taking medications. Therefore, few or no instances of GB reported by the participants of our sample could be attributed to adverse drug reactions.

The threshold value of smoking more than 10 cigarettes per day that was used in the present study is commonly adopted as a risk factor for periodontal disease, and it remains in the 2017 classification system of periodontal diseases [2,40]. We found that SRGB prevalence was associated with smoking status (logistic model), with a lower prevalence in the group smoking more than 10 cigarettes per day. This is in line with the fact that smokers show reduced signs of gingival inflammation and lower GB prevalence compared to nonsmokers; the gingival vasoconstriction induced by the action of nicotine could suppress the inflammatory response that accounts for impaired wound healing [41,42,43]. Nevertheless, certain authors found that BoP was more prevalent in smokers [44,45], but noticed that plaque levels were higher among smokers, and assumed that the vasoconstrictive properties of nicotine may only contribute to a delay in observed GB. In the latter study, smoking status was a significant factor for GB after adjusting for plaque and periodontal probing depth. In the present study, plaque levels could not be measured, but the frequency of toothbrushing was taken into account, as it is thought to be linked to plaque accumulation. Only the frequency of toothbrushing (but neither the type of toothbrush nor the technique) was significantly associated with SRGB. This result is in accordance with previous studies showing that less frequent toothbrushing was significantly associated with a higher frequency of SRGB [28].

Stress and anxiety are known predisposing factors for periodontal disease [46], including necrotizing ulcerative gingivitis and GB [47]. Patients with higher anxiety scores showed significantly more bleeding on probing than patients with lower scores [5]. Stressors and anxious responses to psychological stressors may modulate the immune response to bacteria and influence the progression of gingivitis and periodontitis [48]. Interestingly, smoking (OR = 0.26) and stress (OR = 1.78) were significantly associated with the GB [49]. Academic stress may lead to increased plaque levels and gingival index in students [50,51]. However, in the case of self-assessed GB, anxiety may lead to an overestimation of most symptoms, including GB. Thus, a higher prevalence of SRGB found among anxious subjects may partly derive from this bias in the assessment process. 

The representability of the sample at a national level is relatively satisfactory since the distributions according to the main sociodemographic factors are comparable between the general French population and the selected samples drawn from the four French cities. The outcome variables are based on self-reporting and not on a clinical examination by a practitioner. Systematic reviews evaluating the validity of a questionnaire and/or self-reported measures for periodontal diseases, including gingivitis screening, have concluded that self-perceived pathologies were somewhat more specific (SP) than sensitive (SN) [11,12]. The same results were recently found by Ueno et al. and Saka-Herran et al. [14,52], with higher specificity than sensitivity, even if sensitivity was remarkably high in the latter study. For a given item, sensitivity and specificity depend on the cut-off used to define the bleeding and on how the gingival bleeding is evaluated, which is specific to each study. For example, Buhlin et al. found an SN of 42% and an SP of 76% for the question “Do your gums usually bleed?”, but the cut-off point for gingivitis as regards BoP was set at 30% for the middle-aged group and 50% for the older-aged group, which is not the actual definition of gingivitis as reported in the new classification of 2018 [34]. “Bleeding during toothbrushing” reported by patients in the Kallio study was significantly correlated with the percentage of sites bleeding on probing, showing an agreement of 74%, with a kappa of 0.27, a specificity of 72%, and a sensitivity of 24% [30]. Gilbert and Nuttall presented similar conclusions regarding the comparison of a self-reported and clinical evaluation of GB: a high SP (86–88%) and low SN (19–35%) were found, showing that many people with clinically detected bleeding were unaware of any bleeding after toothbrushing [19]. Nevertheless, the self-evaluation of gingival bleeding can be easily accomplished by anyone and is likely to be a useful diagnostic indicator of gingivitis. This self-evaluation could be an interesting tool for detecting periodontal diseases, as GB is one of the primary symptoms of periodontitis, and may not only facilitate the implementation of lower-cost epidemiological surveys but also improve and reinforce oral hygiene. Indeed, as shown in the Walsh et al. study, a test group having carried out self-assessment of GB with toothbrushing and toothpicks showed a significant reduction of bleeding on probing sites after three months in comparison with a control group [53].

## 5. Conclusions

Until now, there have been no available data at a national level in France regarding SRGB. More than half of the interviewed population (63.2%) experienced GB at least once, spontaneously or after brushing. In the multinomial regression, many parameters were found to be associated with SRGB: nonsmoking young people with high anxiety and toothbrushing twice per day or less reported GB more often. Detecting, monitoring, and managing GB, which is one of the early signs of gingivitis, could be a primary preventive strategy for limiting the development of periodontal diseases such as periodontitis. Therefore, patient awareness regarding the impact of GB may play an important role in public health dentistry.

## Figures and Tables

**Table 1:** Sociodemographic characteristics of all the respondents and respondents with self-reported gingival bleeding (GB).

Sociodemographic and Dental Characteristics	Total N = 794		Preventive Medicine		Railway Station or Mall		
			N = 399 (50.3%)		N = 395 (49.7%)		
	N	%	N	%	N	%	*p* *
**Age (class)**							0.039
18–40 years	367	46.2	198	54.0	169	46.0	
41–60 years	267	33.6	134	50.2	133	49.8	
>60 years	160	20.2	67	41.9	93	58.1	
**Sex**							0.40
Male	376	47.4	183	48.7	193	51.3	
Female	418	52.6	216	51.7	202	48.3	
**Smoker**							0.0032
No	558	70.3	266	47.7	292	52.3	
Yes <10 cig/day	130	16.4	83	63.8	47	36.2	
>10 cig/day	106	13.4	50	47.2	56	52.8	
**Education level (class)**							0.0001
None/low level	280	35.3	169	60.4	111	39.6	
Medium level	143	18.0	61	42.7	82	57.3	
High level	371	46.7	169	45.6	202	54.4	
Occupation (class)							0.001
None, unemployed, student, other	233	29.3	123	52.8	110	47.2	
Retired	141	17.8	55	39.0	86	61.0	
Manual worker, employee, artisan, retailer, administrative	301	37.9	171	56.8	130	43.2	
Teachers, liberal profession, executive	119	15.0	50	42.0	69	58.0	
**Type of toothbrush**							0.082
Unknown	84	10.6	44	52.4	40	47.6	
Soft	272	34.3	120	44.1	152	55.9	
Medium	328	41.3	173	52.7	155	47.3	
Hard	110	13.9	62	56.4	48	43.6	
**Technique of toothbrushing (class)**							0.085
Horizontal	151	19.0	75	49.7	76	50.3	
Vertical	242	30.5	107	44.2	135	55.8	
Circular brushing	172	21.7	89	51.7	83	48.3	
Combination of at least 2 techniques	229	28.8	128	55.9	101	44.1	
**Frequency of toothbrushing (class)**							0.038
≤1 time/day	172	21.7	101	58.7	71	41.3	
2 times/day	466	58.7	226	48.5	240	51.5	
3 times/day	156	19.6	72	46.2	84	53.8	

Notes: Cig/day: cigarettes per day; low level: primary education (8 years or less); medium level: some secondary education (9–11 years); high level: completed secondary education (12 years or more); PAA: platelet antiaggregant; AVK: anti-vitamin K; NSAID: nonsteroidal anti-inflammatory drug; * chi-square test.

**Table 2:** Overall prevalence and type of self-reported gingival bleeding among the population.

Self-Reported Gingival Bleeding *		N	%
No		292	36.8
Yes		502	63.2
After toothbrushing		466	58.7
Spontaneously		33	4.5
**Frequency of self-reported gingival bleeding (class)**			
No		292	
Yes <1 per month		136	27.1
≥1 per month		155	30.9
≤1 per week		111	22.1
>1 per week		100	19.9
**Self-reported gingival bleeding (women N = 418)**			
No bleeding		140	33.5
Hormonal bleeding **	No	226	54.1
	Yes	52	18.7

Note: * Self-reported gingival bleeding of the people interviewed in all the centers of the four cities; ** hormonal bleeding during pregnancy, menstrual bleeding, and the use of contraceptive pills.

**Table 3:** Prevalence of self-reported gingival bleeding according to the different centers selected in the four French cities.

	Total N = 794		Preventive Medicine N = 399 (50.3%)		Railway and Mall N = 395 (49.7%)		*p* *
	N	%	N	%	N	%	
**Self-reported gingival bleeding**							
No	292	36.8	116	39.7	176	60.3	<0.0001
Yes	502	63.2	283	56.4	219	43.6	
**Frequency of self-reported GB among people bleeding (Class)**							
<1 per month	136	27.1	85	62.5	51	37.5	0.28
≥1 per month	155	30.9	80	51.6	75	48.4	
≤1 per week	111	22.1	64	57.7	47	42.3	
>1 per week	100	19.9	54	54.0	46	46.0	
**Self-reported hormonal bleeding among women with bleeding**							
No	226	81.3	133	58.9	93	41.2	0.09
Yes	52	18.7	24	46.2	28	53.9	

* Chi-square or Fisher’s exact test.

**Table 4:** Characteristics of the population according to the presence of self-reported gingival bleeding.

Sociodemographic Characteristics and Drug Information	Total		Self-Reported Gingival Bleeding				
	N = 794		No N = 292 (36.8%)		Yes N = 502 (63.2%)		
	N	%	N	%	N	%	*p* *
**Age (class)**							<0.0001
18–40 years	367	46.2	107	29.2	260	70.8	
41–60 years	267	33.6	98	36.7	169	63.3	
>60 years	160	20.2	87	54.4	73	45.6	
**Sex**							0.043
Male	376	47.4	152	40.4	224	59.6	
Female	418	52.6	140	33.5	278	66.5	
**Smokers**							0.19
No	558	70.3	196	35.1	362	64.9	
Yes <10 cig/day	130	16.4	49	37.7	81	62.3	
>10 cig/day	106	13.4	47	44.3	59	55.7	
**Education level**							0.78
None/low level	280	35.3	101	36.1	179	63.9	
Medium level	143	18.0	50	35.0	93	65.0	
High level	371	46.7	141	38.0	230	62.0	
**Occupations**							<0.0001
None, unemployed, student, other	233	29.3	68	29.2	165	70.8	
Retired	141	17.8	74	52.5	67	47.5	
Manual worker, employee, artisan, retailer, administrative	301	37.9	96	31.9	205	68.1	
Teacher, liberal profession, executive	119	15.0	54	45.4	65	54.6	
**Medicines with hemorragic risk (oral anticoagulant/PAA/AVK/heparin/NSAID)**							0.87
No	733	92.3	269	36.7	464	63.3	
Yes	61	7.7	23	37.7	38	62.3	
**Anxiolytic or antidepressant drugs**							0.067
No	765	96.3	286	37.4	479	62.6	
Yes	29	3.7	6	20.7	23	79.3	
**Cholesterol-lowering**							0.16
No	749	94.3	271	36.2	478	63.8	
Yes	45	5.7	21	46.7	24	53.3	
**Anti-hypertensive drugs**							0.39
No	719	90.6	261	36.3	458	63.7	
Yes	75	9.4	31	41.3	44	58.7	
**Anxiety (class)**							0.0004
No anxiety	438	55.2	185	42.2	253	57.8	
Moderate	197	24.8	67	34.0	130	66.0	
High/severe	159	20.0	40	25.2	119	74.8	

Note: Cig/day: cigarettes per day; low level: primary education (8 years or less); medium level: some secondary education (9–11 years); high level: completed secondary education (12 years or more); PAA: platelet antiaggregant; AVK: anti-vitamin K; NSAID: nonsteroidal anti-inflammatory drug; * chi-square test.

**Table 5:** Multiple logistic regression of covariables associated with self-reported gingival bleeding.

Covariables	Odds-Ratio Confidence Limits	*p*-Value
**Occupation**		<0.0001
None, unemployed, student, other	2.1 [1.3; 3.4]	
Retired	0.7 [0.4; 1.1	
Manual worker, employee, artisan, retailer, administrative	1.8 [1.1; 2.8]	
Teacher, liberal profession, executive	1	
**Smoking status**		0.0012
No	1	
Yes	0.6 [0.4; 0.8]	
**Brushing frequency**		0.0047
≤2 times/day	1.7 [1.2; 2.5]	
3 times/day	1	
**Anxiety level**		0.0031
No anxiety	1	
Moderate	1.3 [0.9; 1.9]	
High/severe	2.0 [1.3; 3.1]	

**Table 6:** Multinomial logistic regression of covariables associated with self-reported gingival bleeding.

Covariables	Spontaneous GB vs. Lack GB Odds-Ratio Confidence Limits	Toothbrushing GB vs. Lack GB Odds-Ratio Confidence Limits	p-Value
**Occupation**			<0.0001
None, unemployed, student, other	4.0 [0.8; 19.3]	2.0 [1.3; 3.3]	
Retired	1.8 [0.3; 9.6]	0.6 [0.4; 1.1]	
Manual worker, employee, artisan, retailer, administrative	5.1 [1.1; 22.9]	1.7 [1.1; 2.6]	
Teacher, liberal profession, executive	1	1	
**Smoking status**			0.0036
No	1	1	
Yes	0.8 [0.4; 1.7]	0.6 [0.4; 0.8]	
**Brushing frequency**			0.0065
≤2 times/day	5.5 [1.3;23.7]	1.6 [1.1;2.3]	
3 times/day	1	1	
**Anxiety level**			0.0130
No anxiety	1	1	
Moderate	0.9 [0.4;2.4]	1.3 [0.9;1.9]	
High/severe	2.4 [1.1; 5.6]	2.0 [1.3; 3.1]	

## Supplementary Materials

The following are available online at https://www.mdpi.com/1660-4601/17/22/8563/s1, Table S1. Comparison of the characteristics of people from each 4 cities, Table S2: Prevalence of self-reported gingival bleeding according to the type of toothbrush and the toothbrushing frequency.

## Appendix A. Material and Method-Interview

The questionnaire comprised 25 items: socioeconomic status (4 items: age, gender, occupation, education level), tobacco use, toothbrushing habits (3 items: toothbrush type, technique, frequency), medication (10 items: drug families) and anxiety level (4 items). GB comprised 3 items: kind of bleeding (no, spontaneous, after brushing), bleeding frequency (less than once per month, once per month, once per week, more than once per week), hormonal bleeding (no, during pregnancy, during menstruation, contraceptive pill). Age was categorized into 3 groups: 18-40 years, 41-60 years, more than 60 years. Occupational categories were classified into 4 groups: unemployed/students, retired, workers/retailers and teachers/senior executives. Anxiety level was assessed by the French version of the Corah’s Dental Anxiety Scale (DAS) which has been shown to be one of the more inclusive, highly validated, and reliable scales [54,55]. This scale provides a score ranging between 4 and 20, which is correlated to the fear perceived by the patient at the dentist’s office. Individuals were considered as not anxious for a DAS score <9, moderately anxious for a DAS score between 9 and 12, and highly/severely anxious for a DAS score ≥13. Four dental students separately conducted the interviews and filled in the questionnaires in their respective cities every day from 9 am to 7 pm.

The participants were free to refuse the interview and the recruitment was based on a convenience sample. Participation was voluntary, anonymous, and without any compensation. Anonymity was guaranteed at all phases of data collection and analyses. Each interview lasted approximately 5 min per subject.

To be included in this survey, the subjects had to be at least 18 years-old, have at least 20 remaining teeth and accepting to respond to the questions after explanation of the survey’s purpose.

## Appendix B. Statistical Methods

The sample size was calculated with respect to the multiple logistic regression model which was used in the statistical analyses. According to Peduzzi et al. [56], the minimum number of cases to include is equal to N = 10 k/p, k being the number of covariates in the regression model and p being the smallest of the proportions of positive or negative cases in the population (here the prevalence of GB). With k = 20 (the number of covariates in the regression model) and p = 0.30 (the most common prevalence of GB found in the literature), N = 666. Considering the proportion of potentially erroneous or incomplete questionnaires, the estimated sample size was 800. Therefore, we included 200 subjects in each of the four cities.

## Appendix C. Results: Characteristics of the Population

Chi-squared tests were conducted to compare these distributions. There was no difference between the sample and the French population for age distribution [16]: 46.2% vs 43.4% for the 18–40 years, 33.6% vs. 34.7% for the 41–60 years and 20.2% vs. 21.9% for the >60 years age group. There were 52.6% of female in the study group and 52.5% in the French adult population. There was also no significant difference for the occupational categories: retailers (5.4% vs. 6.0%), executives (17.0% vs. 16.1%), administrative (23.4% vs. 24.6%), employees (29.2% vs. 28.5%), manual workers (18.8% vs. 21.9%), others (5.5% vs. 1.4%).

## Appendix D. Results Self-Reported Gingival Bleeding Prevalence

No significant difference has been observed between self-reported GB frequency of people from the four cities. The overall bleeding was 65.0% (N = 131) for Montpellier,62.0% (N = 125) for Nancy, 58.7% (N = 118) for Paris and 66.3% (N = 128) for Rennes (p = 0.40) (Data not shown). Characteristics of respondents differed significantly between the four regions (Table S1). However, the region was not associated with the outcome, i.e. gingival bleeding, and then, may not be considered as a confounding factor. So, no adjustment was performed on the region in the multivariable analysis to identify risk factors of SRGB.

## Appendix E. Relations between Self-Reported GB and the Type and Frequency of Toothbrushing

Neither the type of toothbrush nor the technique of toothbrushing have been significantly associated with self-reported GB. Only the frequency of toothbrushing is statistically correlated, and after mixing in the same category ‘less than twice a day’ and ‘twice a day’ (sometimes people state that they brush their teeth twice a day, even when actually it is less often), the bleeding prevalence was significantly associated with the brushing frequency (p < 0.008) (Data not shown).

## References

[1] Marcenes W., Kassebaum N.J., Bernabé E., Flaxman A., Naghavi M., Lopez A., Murray C.J. (2013). Global burden of oral conditions in 1990–2010: A systematic analysis. J. Dent. Res..

[2] Canton J.G., Armitage G., Berglundh T., Chapple I.L.C., Jepsen S., Kornman K.S., Mealey B.L., Papapanou P.N., Sanz M., Tonetti M.S. (2018). A new classification scheme for periodontal and peri-implant diseases and conditions-Introduction and key changes from the 1999 classification. J. Periodontol..

[3] Lang N.P., Tonetti M.S. (2003). Periodontal risk assessment (PRA) for patients in supportive periodontal therapy (SPT). Oral Health Prev. Dent..

[4] Zini A., Sgan-Cohen H.D., Marcenes W. (2011). Socio-economic position, smoking, and plaque: A pathway to severe chronic periodontitis. J. Clin. Periodontol..

[5] Guentsch A., Stier C., Raschke G.F., Peisker A., Fahmy M.D., Kuepper H., Schueler I. (2017). Oral health and dental anxiety in a German practice-based sample. Clin. Oral Investig..

[6] Kitagawa M., Kurahashi T., Matsukubo T. (2017). Relationship between general health, lifestyle, oral health and periodontal disease in adults: A large cross-sectional study in Japan. Bull. Tokyo Dent. Coll..

[7] Bondon-Guitton E., Mourgues T., Rousseau V., Cousty S., Cottin J., Drablier G., Micallef J., Montastruc J.L. (2017). Gingival bleeding, a possible “serious” adverse drug reaction: An observational study in the French pharmacovigilance database. J. Clin. Periodontol..

[8] Bourgeois D., Hescot P., Doury J. (1997). Periodontal conditions in 35–44-yr-old adults in France, 1993. J. Periodontal Res..

[9] Bourgeois D., Doury J., Hescot P. (1999). Periodontal conditions in 65–74-year-old adults in France, 1995. Int. Dent. J..

[10] Bourgeois D., Bouchard P., Mattout C. (2007). Epidemiology of periodontal status in dentate adults in France, 2002–2003. J. Periodontal Res..

[11] Blicher B., Joshipura K., Eke P. (2005). Validation of self-reported periodontal disease: A systematic review. J. Dent. Res..

[12] Ramos R.Q., Bastos J.L., Peres M.A. (2013). Diagnostic validity of self-reported oral health outcomes in population surveys: Literature review. Rev. Bras. Epidemiol..

[13] Carra M.C., Gueguen A., Thomas F., Pannier B., Caligiuri G., Steg P.G., Zins M., Bouchard P. (2018). Self-report assessment of severe periodontitis: Periodontal screening score development. J. Clin. Periodontol..

[14] Saka-Herrán C., Jané-Salas E., González-Navarro B., Estrugo-Devesa A., López-López J. (2020). Validity of a self-reported questionnaire for periodontitis in Spanish population. J. Periodontol..

[15] Vandenbroucke J.P., Von Elm E., Altman D.G., Gøtzsche P.C., Mulrow C.D., Pocock S.J., Poole C., Schlesselman J.J., Egger M. (2007). Strengthening the Reporting of Observational Studies in Epidemiology (STROBE). Epidemiology.

[16] The PLOS Medicine Editors (2014). Observational Studies: Getting Clear about Transparency. PLoS Med..

[17] (2018). Institut National des Statistiques et des Etudes Economiques (INSEE). https://statistiqueslocales.insee.fr/#c=indicator&i=pop_depuis_1876.pop&i2=pop_depuis_1876.dens&s=2016&s2=2016&view=map2.

[18] Pinelli C., de Castro Monteiro L.L. (2007). Reproducibility and validity of self-perceived oral health conditions. Clin. Oral Investig..

[19] Gilbert A.D., Nuttall N.M. (1999). Self-reporting of Periodontal Health Status. Br. Dent. J..

[20] Genco R.J., Falkner K.L., Grossi S., Dunford R., Trevisan M. (2007). Validity of Self-Reported Measures for Surveillance of Periodontal Disease in Two Western New York Population-Based Studies. J. Periodontol..

[21] Rolstad S., Adler J., Rydén A. (2011). Response burden and questionnaire length: Is shorter better? A review and meta-analysis. Value Health.

[22] Sahlqvist S., Song Y., Bull F., Adams E., Preston J., Ogilvie D., iConnect Consortium (2011). Effect of questionnaire length, personalisation and reminder type on response rate to a complex postal survey: Randomised controlled trial. BMC Med. Res. Methodol..

[23] Taylor G.W., Borgnakke W.S. (2007). Self-reported periodontal disease: Validation in an epidemiological survey. J. Periodontol..

[24] Dietrich T., Stosch U., Dietrich D., Schamberger D., Bernimoulin J.P., Joshipura K. (2005). The accuracy of individual self-reported items to determine periodontal disease history. Eur. J. Oral Sci..

[25] Vered Y., Sgan-Cohen H.D. (2003). Self-perceived and clinically diagnosed dental and periodontal health status among young adults and their implications for epidemiological surveys. BMC Oral Health.

[26] Yamamoto T., Koyama R., Tamaki N., Maruyama T., Tomofuji T., Ekuni D., Yamanaka R., Azuma T., Morita M. (2009). Validity of a questionnaire for periodontitis screening of Japanese employees. J. Occup. Health.

[27] Nadanovsky P., Dos Santos A.P.P., Bloch K.V. (2018). Prevalence of self-reported gingival bleeding in a, representative sample of the Brazilian adolescent population. J. Clin. Periodontol..

[28] Lintula T., Laitala V., Pesonen P., Sipilä K., Laitala M.L., Taanila A., Anttonen V. (2014). Self-reported oral health and associated factors in the North Finland 1966 birth cohort at the age of 31. BMC Oral Health.

[29] Kim H.N., Jang Y.E., Kim C.B., Kim N.H. (2018). Socioeconomic status and self-reported periodontal symptoms in community-dwelling individuals: Data from the Korea Community Health Surveys of 2011 and 2013. Int. Dent. J..

[30] Kallio P., Uutela A., Nordblad A., Alvesalo I., Murtomaa H., Croucher R. (1997). Self-assessed bleeding and plaque as methods for improving gingival health in adolescents. Int. Dent. J..

[31] Romano F., Perotto S., Bianco L., Parducci F., Mariani G.M., Aimetti M. (2020). Self-Perception of Periodontal Health and Associated Factors: A Cross-Sectional Population-Based Study. Int. J. Environ. Res. Public Health.

[32] Bruers J.J., van Rossum G.M. (1998). Dental care frequency of female patients in the Netherlands. Ned. Tijdschr. Tandheelkd..

[33] Zachariasen R.D. (1991). Ovarian hormones and gingivitis. J. Dent. Hygiene.

[34] Buhlin K., Gustafsson A., Andersson K., Håkansson J., Klinge B. (2002). Validity and Limitations of Self-Reported Periodontal Health. Community Dent. Oral Epidemiol..

[35] Ebersole J.L., Graves C.L., Gonzalez O.A., Dawson D., Morford L.A., Huja P.E., Hartsfield J.K., Huja S.S., Pandruvada S., Wallet S.M. (2016). Aging, inflammation, immunity and periodontal disease. Periodontology.

[36] Kawachi I., Kennedy B.P. (1999). Income inequality and health: Pathways and mechanisms. Health Serv. Res..

[37] Braveman P.A., Cubbin C., Egerter S., Chideya S., Marchi K.S., Metzler M., Posner S. (2005). Socioeconomic status in health research: One size does not fit all. JAMA.

[38] Tack D.A., Rogers R.S. (2002). Oral drug reactions. Dermatol. Ther..

[39] Bascones-Martinez A., Muñoz-Corcuera M., Bascones-Ilundain C. (2015). Side effects of drugs on the oral cavity. Med. Clin..

[40] Dietrich T., Ower P., Tank M., West N.X., Walter C., Needleman I., Hughes F.J., Wadia R., Milward M.R., Hodge P.J. (2019). Periodontal diagnosis in the context of the 2017 classification system of periodontal diseases and conditions-implementation in clinical practice. Br. Dent. J..

[41] Müller H.P., Heinecke A., Eger T. (2000). Site-specific association between supragingival plaque and bleeding upon probing in young adults. Clin. Oral Investig..

[42] Mullally B.H. (2004). The influence of tobacco smoking on the onset of periodontitis in young Persons. Tob. Induc. Dis..

[43] Al-Bayaty F.H., Baharuddin N., Abdulla M.A., Ali H.M., Arkilla M.B., Al-Bayaty M.F. (2013). The influence of cigarette smoking on gingival bleeding and serum concentrations of haptoglobin and Alpha 1-Antitrypsin. Biomed. Res. Int..

[44] Müller H.P., Stadermann S., Heinecke A. (2002). Gingival recession in smokers and non-smokers with minimal periodontal disease. J. Clin. Periodontol..

[45] Müller H.P., Stadermann S. (2006). Multivariate multilevel models for repeated measures in the study of smoking effects on the association between plaque and gingival bleeding. Clin. Oral Investig..

[46] Akcali A., Huck O., Tenenbaum H., Davideau J.L., Buduneli N. (2013). Periodontal diseases and stress: A brief review. J. Oral Rehabil..

[47] Herrera D., Alonso B., de Arriba L., Santa Cruz I., Serrano C., Sanz M. (2014). Acute periodontal lesions. Periodontology.

[48] Breivik T., Thrane P.S., Murison R., Gjermo P. (1996). Emotional stress effects on immunity, gingivitis and periodontitis. Eur. J. Oral Sci..

[49] Hugo F.N., Hilgert J.B., Bozzetti M.C., Bandeira D.R., Gonçalves T.R., Pawlowski J., de Sousa M.L. (2006). Chronic stress, depression, and cortisol levels as risk indicators of elevated plaque and gingivitis levels in individuals aged 50 years and older. J. Periodontol..

[50] Deinzer R., Rüttermann S., Möbes O., Herforth A. (1998). Increase in gingival inflammation under academic stress. J. Clin. Periodontol..

[51] Ravishankar T.L., Ain T.S., Gowhar O. (2014). Effect of academic stress on plaque and gingival health among dental students of Moradabad, India. J. Int. Acad. Periodontol..

[52] Ueno M., Shimazu T., Sawada N., Tsugane S., Kawaguchi Y. (2020). Validity of Self-Reported Periodontitis in Japanese Adults: The Japan Public HealthCenter–Based Prospective Study for the Next-Generation Oral Health Study. Asia Pac. J. Public Health.

[53] Walsh M.M., Heckman B.H., Moreau-Diettinger R. (1985). Use of gingival bleeding for reinforcement of oral home care behavior. Community Dent Oral Epidemiol..

[54] Corah N.L. (1969). Development of a dental anxiety scale. J. Dent. Res..

[55] Nicolas E., Collado V., Faulks D., Bullier B., Hennequin M. (2007). A national cross-sectional survey of dental anxiety in the French adult population. BMC Oral Health.

[56] Peduzzi P., Concato J., Kemper E., Holford T.R., Feinstein A.R. (1996). A simulation study of the number of events per variable in logistic regression analysis. J. Clin. Epidemiol..

