# Supplementary material for: Prevalence and Associated Factors of Self-Reported Gingival Bleeding: A Multicenter Study in France
Source: Int J Environ Res Public Health. 2020 Nov 18;17(22):8563. doi: 10.3390/ijerph17228563 (PMC7698919; doi:10.3390/ijerph17228563)
Supplement: Supplementary file 1 [file ijerph-17-08563-s001.pdf]

Table S1. Comparison of the characteristics of people from each 4 cities.

		Total N= 794		Nancy N=200 (25.2%)		Montpellier N=200 (25.2%)		Rennes N=193 (24.3%)		Paris N=201 (25.3%)		
		N	%	N	%	N	%	N	%	N	%	p*
**Age by range**												0.0009
18-40		367	46.2	79	39.5	89	44.5	105	54.4	94	46.8	
41-60		267	33.6	60	30.0	78	39.0	58	30.1	71	35.3	
> 60		160	20.2	61	30.5	33	16.5	30	15.5	36	17.9	
**Sex**												0.2095
Male		376	47.4	102	51.0	83	41.5	90	46.6	101	50.2	
Female		418	52.6	98	49.0	117	58.5	103	53.4	100	49.8	
**Smoker**												0.0006
No		558	70.3	136	68.0	146	73.0	125	64.8	151	75.1	
Yes	<10 cig/j	130	16.4	23	11.5	28	14.0	43	22.3	36	17.9	
Yes	>10 cig/j	106	13.4	41	20.5	26	13.0	25	13.0	14	7.0	
**Education level (class)**												<0.0001
None		280	35.3	88	44.0	75	37.5	74	38.3	43	21.4	
Medium Level		143	18.0	23	11.5	42	21.0	44	22.8	34	16.9	
High Level		371	46.7	89	44.5	83	41.5	75	38.9	124	61.7	
**Occupation (class)**												<0.0001
None,unemployed,student,others		233	29.3	62	31.0	42	21.0	76	39.4	53	26.4	
Retired		141	17.8	58	29.0	22	11.0	33	17.1	28	13.9	
Manual worker, Employee, Artisan, Retailer, Administrative		301	37.9	50	25.0	114	57.0	64	33.2	73	36.3	
Teachers, Liberal profession,executive		119	15.0	30	15.0	22	11.0	20	10.4	47	23.4	
**Self reported gingival bleeding**												0.3404
No		292	36.8	76	38.0	68	34.0	65	33.7	83	41.3	
Yes		502	63.2	124	62.0	132	66.0	128	66.3	118	58.7	

Note: Cig/d: cigarettes per day; Low level: primary education (8 years or less), Medium level: some secondary education (9-11 years), High level: completed secondary education (12 years or more). * Chi-2 test

Table S2. Prevalence of self-reported gingival bleeding according to the type of toothbrush and the toothbrushing frequency.

	Total N= 794		Gingival bleeding No N= 292 (36.8%)		Gingival bleeding Yes N=502 (63.2%)		
	N	%	N	%	N	%	p*
**Type of toothbrush**							0.77
Unknown	84	10.6	28	33.3	56	66.7	
Soft	272	34.3	97	35.7	175	64.3	
Medium	328	41.3	127	38.7	201	61.3	
Hard	110	13.9	40	36.4	70	63.6	
**Toothbrushing techniques**							0.77
Horizontal	151	19.0	52	34.4	99	65.6	
Vertical	242	30,5	91	37.6	151	62.4	
Circular brushing	172	21.7	60	34.9	112	65.1	
Combination of at least 2 techniques	229	28.8	89	38.9	140	61.1	
**Frequency of toothbrushing (class)**							0.025
≤ 1 time/day	172	21.7	60	34.9	112	65.1	
2 times/day	466	58.7	160	34.3	306	65.7	
3 times/day	156	19.6	72	46.2	84	53.8	

Note: * Chi-2.
